# The dilemma of trauma-focused therapy: effects of imagery rescripting on voluntary memory

**DOI:** 10.1007/s00426-022-01746-z

**Published:** 2022-11-05

**Authors:** Maximilian Ganslmeier, Anna E. Kunze, Thomas Ehring, Larissa Wolkenstein

**Affiliations:** Department of Psychology, Ludwig-Maximilians-Universität München, Leopoldstraße 13, 80802 Munich, Germany

## Abstract

Trauma-focused imagery-based interventions are suspected to alter or even distort declarative voluntary memory of a traumatic event, especially if they involve the active modification of imagery, e.g., as used in imagery rescripting (ImRs). However, systematic research is lacking so far. To investigate whether ImRs modifies voluntary memory of a standardized autobiographical aversive event (Trier Social Stress Test) (Session 1), healthy participants (*N* = 100) were randomly assigned to either an intervention condition receiving one session of ImRs or to a no-intervention control condition (NIC) (Session 2). Voluntary memory was examined using a free recall (Sessions 2 and 3) and a cued recall (Sessions 3 and 4). Although voluntary memory tended to deteriorate over time, contrary to expectations, this effect was not associated with ImRs. Remarkably, the number of correct details in free recall even improved in ImRs but not in NIC. This challenges the view that ImRs alters voluntary memory.

**Supplementary Information:**

The online version contains supplementary material available at 10.1007/s00426-022-01746-z.

## Introduction

Post-traumatic stress disorder (PTSD) is a prevalent and disabling disorder triggered by traumatic experiences, such as experiencing physical or sexual violence, and often requires psychological treatment (McCart et al., 2010). If survivors decide to sue the offender during or after therapy, the credibility of their testimony may be evaluated by eyewitness experts (Otgaar et al., 2017), and can include an assessment of whether the testimony could constitute a *false memory*, i.e., a memory that feels subjectively to be based on a true event but cannot be attributed to an actual experience (Brainerd et al., 2008). Since the 1990s, it has been commonly assumed that psychological interventions may distort declarative memory and might even be involved in the creation of false memories (Lindsay & Read, 1994; Porter et al., 2012). Trauma-focused cognitive behavioral therapy is the current gold standard treatment for PTSD and often includes imagery-based interventions (Courtois et al., 2017; Cusack et al., 2016; Weber et al., 2021) which have been suggested to carry a risk of distorting memory or even inducing (false) memories (Brainerd & Reyna, 2005; Ridley et al., 2012). Since narratives of false and true memories do not systematically differ from each other (Blandón-Gitlin et al., 2009), in the absence of objective information there is no evidence-based method to reliably distinguish between them. Thus, it is frequently assumed by courts and their expert witnesses that the credibility of a trauma survivor who has received imagery-based trauma-focused treatment can no longer be determined and is, therefore, regarded as potentially impaired (Finer, 1996; Otgaar et al., 2019). Hence, lawyers often advise victims not to begin trauma-focused therapy before criminal proceedings are concluded (Bublitz, 2020). This leaves patients and therapists with the dilemma of having to choose between a patient’s psychological well-being and the maintenance of credibility and the associated likelihood of legal success (Bublitz, 2020).

The assumption that imagery-based trauma-focused interventions can distort the declarative *voluntary*^1^ memory of a traumatic event – which includes knowledge of facts and trauma episodes that are recalled deliberately when one decides to recount the trauma (Visser et al., 2018) – is based on evidence that human memory is dynamic. After encoding and consolidation, a memory becomes temporarily unstable upon reactivation (Kindt et al., 2009; Nader et al., 2000), allowing new information to be integrated into the existing memory trace (Moscovitch et al., 2005). During this reconsolidation phase, the content of the memory might temporarily be susceptible to interference, as factually incorrect information could also be integrated (Scully et al., 2017).

Analogue studies with healthy samples have shown that imagination may be particularly potent in altering memories. In these studies, three main experimental paradigms have been used to simulate possible therapy-induced biases in autobiographical memory: (1) imagination inflation paradigm, in which participants were asked to repeatedly imagine events that they actually have not experienced or (2) false feedback paradigm, in which participants are given false information (e.g., manipulated photos or videos) indicating that they likely experienced an event or (3) memory implantation paradigm, in which the presumed occurrence of an event that did not happen is supported, for example, by false statements by family members (Brewin & Andrews, 2017). Both familiar and usual (e.g., rest on the fire hydrant) as well as bizarre and unusual (e.g., shake hands with the fire hydrants) events were used. Afterwards participants were asked to rate how likely the event has occurred. Results show that imagery can induce and increase subjective confidence that imagined events have actually taken place (Goff & Roediger, 1998; Nash et al., 2009; Seamon et al., 2009; Thomas & Loftus, 2002). Even when participants were warned about the interfering effects of imagination in advance, imagery still increased the false confidence that certain actions had been performed (Nash et al., 2009). The proposed mechanism for this effect has been assumed to be that imagining an event (in all sensory modalities) is experienced and processed in a manner very similar to the sensory-perceptual representation of an actual event, including an overlap in activated brain areas (Holmes & Mathews, 2010).

Based on these findings from basic memory research, expert witnesses have proposed that imagery-based psychological treatment can have the same effect and can, therefore, result in altered or even false memories that are experienced as genuine experiences (Volbert & Steller, 2014). This may be particularly true for interventions such as imagery rescripting (ImRs), which is a promising intervention used to treat maladaptive and traumatic memories (Arntz & Weertman, 1999; Holmes et al., 2007). Therefore, patients are instructed to imagine counterfactual events, i.e., changing the traumatic event into a more benign and less distressing mental image by integrating new information and helpful perspectives (Smucker et al., 1995). Specifically, during ImRs the original memory is first reactivated, which makes it accessible for modification (Arntz, 2012). In a second step, new information that has not happened in reality is actively integrated into the mental image of the memory (Arntz, 2012; Smucker et al., 1995). For example, a PTSD patient may rewrite memories of a sexual assault into a new script that involves successfully defending against the offender or rescuing the victim. ImRs has been shown to be effective in reducing symptom severity in PTSD (Morina et al., 2017).

When comparing imagery-based psychological interventions, such as ImRs, with the experimental manipulations used in the basic memory studies described above (Goff & Roediger, 1998; Nash et al., 2009; Seamon et al., 2009; Thomas & Loftus, 2002), it becomes clear that both indeed include strategies to actively modify memory representations. However, a number of differences are also noteworthy. First, in the analogue studies, memory traces of very short, personally non-relevant events are typically manipulated, whereas ImRs is applied to autobiographical memories of highly emotional aversive and/or traumatic events. As there is an association between emotional intensity during retrieval and strength of autobiographical memories (i.e., tend to be remembered longer, with greater vividness and a greater sense of recollection) (Talarico et al., 2004), this could be crucial. Second, in analogue studies participants are kept unclear about the goal of the manipulation, and the setting is deliberately designed to make it difficult to distinguish between the original and the altered experience. Additionally, instructions often explicitly requested additional details (i.e., imagining events that supposedly took place) or suggested a fictional context (i.e., false testimony of family members or faked photos). In ImRs, however, the integration of new information into the memory is made very salient, i.e., when entering the rescripting phase, the patient is informed that imagery is now used to deviate from the original memory. Besides, patients mainly decide for themselves what they imagine to change the meaning and/or the emotional experience of the memory in order to reduce the intrusive involuntary re-experiencing. Unlike basic memory studies, ImRs does not necessarily add plausible or similar information that might make it difficult for subjects to distinguish between imagined and experienced content because of the similarity in content.

Despite the procedural differences described above, it remains unclear whether ImRs can inadvertently affect patients’ factual knowledge and/or voluntary recollection of the original aversive event. So far, only two studies have addressed this issue. Using an aversive film as trauma analogue, both studies found that ImRs did not impair factual knowledge of the film when compared to active (i.e., positive imagery of a personal, pleasant experience) (Hagenaars & Arntz, 2012) or no-intervention control conditions (Siegesleitner et al., 2019). However, these results are limited in that the studies did not primarily aim to examine the effects of ImRs on voluntary memory and, therefore, lack methodological rigor to draw conclusions about such effects.

The main goal of the current study was to use an experimental analogue design to investigate to what extent ImRs changes autobiographic voluntary memory. In contrast to the aforementioned analogue studies testing the effects of ImRs on selected variables assessing voluntary memory as secondary outcomes (Hagenaars & Arntz, 2012; Siegesleitner et al., 2019), this is the first study directly addressing this research question. The methodology was adapted accordingly. First, although the trauma film paradigm has proven to effectively induce analogue PTSD symptoms (James et al., 2016), the autobiographical quality of the memory is missing when using a film as the stressor (Dibbets & Schulte-Ostermann, 2015). Hence, we used an adapted version of the Trier Social Stress Test (TSST) to induce a standardized but aversive autobiographical experience. Second, voluntary memory was assessed more comprehensively (e.g., using a larger number of cued recall items; adding a free recall task). Third, in addition to short- and mid-term effects we added a three-month follow-up to additionally investigate long-term memory changes. Lastly, the interval between aversive autobiographical experience and intervention was expanded to ensure sufficient time for consolidation.

Based on the theoretical ideas and empirical findings underlying current legal practice, we hypothesized that ImRs (compared to a no-intervention control) would lead to more false details and less details recalled correctly.

## Methods

### Overview

**Fig. 1:**
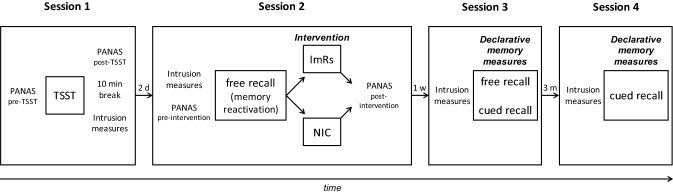
Experimental procedure. *PANAS* Positive and Negative Affect Schedule (Krohne et al., 1996); *TSST* Trier Social Stress Test (Kirschbaum et al., 1993); *ImRs* intervention condition: participants received ImRs as an intervention, *NIC* no-intervention control condition: participants waited 15 min in front of the laboratory

The study comprised four sessions (see Fig. 1). During Session 1, participants completed the TSST. Session 2 followed two days later and included the free recall and the intervention (ImRs vs. NIC). One week later (Session 3), voluntary memory was measured by a second free recall and a first cued recall. After 3 months (Session 4) the cued recall was repeated. To avoid carry-over effects between the tasks, cued recall was only assessed after the completion of both free recalls. The first three sessions were conducted in the laboratory. For Session 4, participants were contacted via e-mail and asked to fill in an online questionnaire (using the online survey software *Unipark*). Due to the online format, we could not repeat the free recall in Session 4.

### Participants

Participants were recruited via announcements on social media, a student e-mail newsletter and public postings. We included university students meeting the following inclusion criteria: (1) age between 18 and 30 years, and (2) fluency in German. In addition, the following exclusion criteria were applied: (1) current psychological disorder (German version of the Mini-International Neuropsychiatric Interview [M.I.N.I.]; Ackenheil et al., 1999) or severe neurological disorder, (2) current psychological treatment, (3) consumption of illegal drugs within the past 3 days, or (4) alcohol consumption of more than three glasses of beer, wine, cocktails or hard liquor within the last 24 h before the experiment. A total of 124 students were assessed for eligibility. Sixteen participants had to be excluded. In addition, six participants dropped out after Session 1, and two dropped out after Session 2. Thus, total sample size was *N* = 100 (71% female; age: *M* = 22.18, *SD* = 3.05). Participants were randomly allocated to one of two experimental conditions: ImRs (*n* = 50) or no-intervention control (NIC) (*n* = 50).

An a priori power analysis was carried out with G*Power (Faul et al., 2009) for sample size planning. Based on prior research (e.g., Garry et al., 1996; Horselenberg et al., 2000) we assumed the effect of imagery on voluntary memory to be of medium size (*f* = 0.25). With *α* = 0.05 and a statistical power of 0.80, it was necessary to enroll 34 participants to detect a Condition $\times$ Time interaction and 98 participants to detect a main effect of Condition or Time on voluntary memory as measured by free and cued recall (2 [Condition] × 2 [Time] ANOVAs).

Participants provided written informed consent and were reimbursed with either €8 per hour or course credits. The study was approved by the local Research Ethics Committee (66_Wolkenstein_b).

### Materials

#### Trier Social Stress Test

We used an adapted version of the Trier Social Stress Test (TSST; Kirschbaum et al., 1993) to induce a negative autobiographical memory (van der Zweerde, 2014). The TSST lasted approximately 15 min and comprised three tasks in front of a committee consisting of two females. First, participants were instructed to imagine having a job interview for a position they would really like to have. They had 3 min to prepare a presentation about their strengths and weaknesses to show why they are the perfect candidate for the position. Afterwards, the committee members entered the room, sat down at a table and asked the participants to start their presentation, which then lasted 5 min. Second, participants were asked to do an arithmetic task (counting backwards from 1310 in steps of 13) for 3 min. As soon as participants made a mistake, they were interrupted and asked to start over. Third, they were asked to sing out loud the German version of the four-verse children's song *All my little ducklings*.

In order to enable voluntary memory tests later-on, members of the committee wore standardized clothing and followed a standardized protocol, specifying when to take notes, when to interrupt participants and how to provide standardized negative feedback (e.g., asking them to speak louder, count faster, sing more melodiously). They maintained a serious facial expression throughout the procedure. Furthermore, participants were told that the interview would be recorded on camera and that the committee was trained in the analysis of non-verbal behavior (neither of these elements was actually true and only told to participants to increase their stress induction).

#### Intervention

*Imagery rescripting (ImRs)* Since ImRs usually involves memory reactivation first, in this design the free recall (see Measures section) immediately preceding ImRs was used for memory reactivation in order to initiate reconsolidation processes (detailed instructions are provided in *Supplementary Material A*). This was followed by the actual ImRs, for which a modified script adapted from the procedure developed by Arntz and Weertman (1999) was used (detailed instructions are provided in *Supplementary Material B*). First, participants were asked to name and briefly describe the most distressing moment during the TSST (hotspot). They were then instructed to close their eyes and reactivate and imagine the scene as vividly as possible from the start of the TSST up to the identified hotspot. They were asked to describe the scene in the present tense and using first person singular, including all sensory, emotional and physical sensations that occurred.

As soon as they had reached the hotspot, the investigators instructed participants to change the script in their imagination in order to achieve an outcome for the scene that was less stressful for them. Participants were told that the changed script could include events that could possibly happen as well as events that are not possible in reality. The investigators accompanied the participant during the imagination exercise by asking in-depth questions, e.g., about the location, people present in the situation, and about sensory perceptions, thoughts and body sensations. As soon as the participants indicated that the outcome of the situation felt comprehensively good to them, ImRs was concluded (duration [minutes]: *M* = 16.32, *SD* = 6.16).

ImRs was recorded on tape and the recording was given to the participants. In analogy to the use of ImRs in psychological treatment, they were instructed to listen to the recording three times before Session 3 (Smucker et al., 1995).

*No-intervention control condition (NIC)* Participants in the control condition did not receive ImRs or any other intervention. They had a 15-min break, in which they sat outside the laboratory room.

### Measures

#### Voluntary memory measures

Voluntary memory was assessed in two ways, using both free recall (in order to assess memory in a broad, complex and individual manner), and cued recall (to assess concrete and specific details).

*Free recall* A free recall task was used to assess possible changes in voluntary memory of the TSST that can be attributed to ImRs. The first free recall task took place in Session 2 and was repeated in Session 3.

Using a standardized script (for detailed instructions, see *Supplementary Material A*), participants were instructed to imagine their experience of the TSST and to verbally report their memory of the TSST as accurately and in as much detail as possible. As in ImRs, they were asked to close their eyes and to describe their experience in the first person singular and in the present tense as if they were experiencing it at that very moment. They were asked to continue describing the scene until they themselves decided that the scene was complete (duration [minutes]: Session 2: *M* = 7.65, *SD* = 4.66; Session 3: *M* = 7.40, *SD* = 4.51). The report was audio recorded, transcribed, and rated to analyze changes in voluntary memory. Changes in the content of voluntary memory were evaluated using a standardized protocol-based rating procedure adapted from Levine et al. (2002) and Jack et al. (2014). For this purpose, each free recall was segmented into informational details (adapted from Levine et al., 2002). A detail was defined as a unique event, observation, or thought, usually expressed as a grammatical clause (i.e., a subject and verb: “*I count backwards*”). Additional information (e.g., “*from 1310*”) was scored separately (e.g., “I count backwards *from 1310*”). There were two broad groups of details as follows: *internal* (specific to the time and place of the TSST, reflecting episodic reexperiencing) and *external* (not specific to the time and place of the TSST, semantic knowledge, repetitions, other details, retrospective appraisals). Internal details were divided into following five exclusive categories: (a) event (e.g., “*I sing…*”), (b) place (e.g., “*to the right of the table*”), (c) time (e.g., “*then*”, “*3 min*”), (d) perception (e.g., “*I see the camera*”), and (e) emotion/thought (e.g., “*I feel angry*”, “*I think that they are really unfriendly*”) (see *Supplementary Material C*).

The ratings were conducted by two independent raters who were blind to the condition. Based on criteria suggested by Koo and Li (2016), interrater reliability, measured by intraclass correlations (ICC), was excellent for ICC_event_ (1, 1) = 0.95, ICC_time_ (1, 1) = 0.97 and ICC_emotion/thought_ (1, 1) = 0.97. It was good for ICC_place_ (1, 1) = 0.82, ICC_perception_ (1, 1) = 0.87 and ICC_externals_ (1, 1) = 0.78.

Following Jack et al. (2014), all internal details belonging to the categories (a) to (d) were rated as *correct* (if they represented details that had been present during the TSST) or *incorrect* (if they represented details that had *not* been present during the TSST). Details that could not be clearly classified as correct or incorrect (due to lack of video recording were evaluated as *possible* (e.g., I leaned on the chair). Details belonging to the emotion/thought category were not rated as correct or incorrect because it could not be objectively judged what participants had been thinking or feeling during the TSST (see *Supplementary Material C*).

Ratings of correct vs. possible vs. incorrect details were also conducted by two independent raters who were blind to the condition. Intraclass correlations (ICC) were excellent for ICC_correct details_ (1, 1) = 0.95 and ICC_possible details_ (1, 1) = 0.93. It was good for ICC_incorrect details_ (1, 1) = 0.75.

Based on the ratings, sum scores were computed for (a) number of correct details, (b) number of incorrect details, and (c) total number of details provided (i.e., number of all internal and external details provided). The latter was used to control for the overall verbal output in subsequent analyses evaluating free recall.

*Cued recall* During Sessions 3 and 4, participants performed a cued recall test. The task was designed to include questions equivalent to those asked during an interrogation by the police (Hermanutz & Schröder, 2015), such as questions about the location (e.g., “*Please name all pieces of furniture, furnishings and living accessories that you remember.*”; correct answers: *table, chairs, lamp, picture, plant, curtain, flipchart:* incorrect answers: *all other, e.g., things that were not in the room, such as folders*), the characteristics of the people involved (e.g., “*What colors were the jury members' tops?*”; correct answers: *black, red;* incorrect answers: *all other*), and the procedure of the TSST (e.g., “*What were you asked to talk about during the presentation?*”; correct answers: *strengths and weaknesses / character traits*); incorrect answers: *all other*). The cued recall comprised a total of 33 questions (for the detailed cued recall, see *Supplementary Material D*). Two independent raters, who were blind to the condition, analyzed and rated the answers. They counted correct answers, incorrect answers, and the answering option “*I do not know*”, which was one answering option of each cued recall item to avoid guessing. Interrater reliability measured by intraclass correlation (ICC) was excellent for the cued recall task: ICC_correct_ (1, 1) = 0.99, ICC_I do not know_ (1, 1) = 0.99, ICC_incorrect_ (1, 1) = 0.97.

#### Manipulation check

Participants filled in the Positive and Negative Affect Schedule (PANAS; German version: Krohne et al., 1996) immediately pre- and post-TSST to assess whether the TSST was experienced as stressful. They also completed the PANAS immediately pre- and post-intervention to assess the effect of the intervention on participants’ mood. The PANAS consists of two scales (positive and negative affect) with ten items each. Participants indicated to what extent each of the affective states applied to them at the moment on a 5-point Likert scale (1 = *not at all*; 5 = *extremely*). Sum scores were calculated for each scale and measurement time. Internal consistencies were acceptable or good for both positive (pre-TSST: *α* = 0.87; post-TSST: *α* = 0.89; pre-intervention: *α* = 0.88; post-intervention: *α* = 0.92) and negative affect (pre-TSST: α = 0.86; post-TSST: *α* = 0.86; pre-intervention: *α* = 0.83; post-intervention: *α* = 0.77).

In all four sessions the occurrence of intrusions related to the TSST was assessed using a questionnaire (intrusion measures) similar to those used in paper tabular diaries (James et al., 2015). Participants indicated how often they had experienced intrusive memories after the TSST, the percentage of time (from 0 to 100) they had experienced them and – if they reported at least one intrusive memory – how stressful, controllable and vivid they experienced the intrusions (0 = *not at all* to 100 = *very much*).

#### Control variables

We assessed general memory performance by the Verbal Learning and Memory Test (VLMT; Helmstaedter & Durwen, 1990) and social anxiety using the Social Interaction Anxiety Scale (SIAS; Stangier et al., 1999) and the Social Phobia Scale (SPS; Stangier et al., 1999) (see Table 1).

**Table 1:** Means (*M*) and standard deviations (*SD*) of sociodemographic and control variables

Variables	Condition		Statistics	*p*	
	ImRs (*n* = 50)	NIC (*n* = 50)			
Sociodemographic variables	*M* (*SD*)	*M* (*SD*)			
Age	22.24 (3.22)	22.12 (2.90)	*t*(98) = − 0.20	0.85	
Number of years of education	15.06 (3.72)	14.65 (3.07)	*t*(98) = − 0.60	0.55	
	%	%			
Gender (female)	72	70	*χ*^2^ (1) = 0.05	1.00	
Lifetime mental illness (yes)^a^	4.2	0.00		0.12^b^	
Lifetime psychotherapeutic/psychiatric treatment (yes)	4	6	*χ*^2^ (1) = 0.44	0.74	
Control variables	*M* (*SD*)	*M* (*SD*)			
Memory: learning performance (VLMT)	57.88 (6.13)	59.70 (8.34)	*t*(98) = 1.24	0.22	
Memory: consolidation (VLMT)	0.62 (2.06)	1.12 (1.98)	*t*(98) = 1.24	0.22	
Memory: recognition (VLMT)	13.90 (1.22)	14.02 (1.42)	*t*(98) = 0.45	0.65	
Social interaction anxiety (SIAS)	20.48 (11.79)	17.94 (11.59)	*t*(98) = − 1.09	0.28	
Social performance anxiety (SPS)	9.20 (7.60)	7.26 (6.59)	*t*(98) = − 1.36	0.18	
Sleep at night after Session 2: sleep duration (in hours)^c^	7.42 (1.51)	7.55 (0.83)	*t*(88) = 0.47	0.64	
Sleep at night after Session 2: sleep quality^c^	1.89 (0.67)	1.91 (0.42)	*t*(88) = 0.15	0.88	
Sleep during last week: sleep duration	7.15 (0.83)	7.27 (0.77)	*t*(98) = 0.77	0.44	
Sleep during last week: sleep quality	2.00 (0.57)	1.94 (0.55)	*t*(98) = − 0.54	0.59	
	%	%			
Sleep at night after Session 2: sleep normality (yes)^c^	89.1	90.9		1.00^b^	
Sleep during last week: sleep normality (yes)	80	76	*χ*^2^ (1) = 0.23	0.81	
Talked to sb. about TSST in the week after (yes)	64	78	*χ*^2^ (1) = 2.40	0.19	
Wrote diary about TSST in the week after (yes)^d^	0.00	0.00			
Ever had similar experience to TSST (yes)	46	42	*χ*^2^ (1) = 0.16	0.84	

*ImRs* intervention condition, *NIC* no-intervention control condition, *f* female, *m* male, *VLMT* Verbal Learning and Memory Test, *SIAS* Social Interaction Anxiety Scale, *SPS* Social Phobia Scale

^a^ImRs (*n* = 49); NIC (*n* = 47)

^b^Fisher’s exact test

^c^ImRs (*n* = 46); NIC (*n* = 44)

^d^No calculation of the test statistic due to the constant value

In addition, participants were asked whether they had gone through the TSST experience repeatedly, e.g., by talking to others or writing a diary (*yes* vs. *no*), and whether they had ever experienced a similar event before (*yes* vs. *no*). Sleep duration and quality after Session 2 and during the last week was also surveyed.

### Procedure

Session 1: After written informed consent was obtained, participants were screened for inclusion and exclusion criteria. Eligible participants were then tested for sociodemographic variables and control variables. This was followed by the PANAS pre-TSST, the TSST, and the PANAS post-TSST. After a 10-min break, the first intrusion questionnaire was administered.^2^

Session 2: When participants returned to the laboratory, they were randomly assigned to one of two conditions (ImRs vs. NIC). Then, sleep quality and duration were assessed, and the intrusion questionnaire was administered for the second time. This was followed by the PANAS (pre-intervention) and the first free recall. After that, participants underwent the ImRs intervention (or the break), followed by the PANAS (post-intervention). At the end of the session, participants in the ImRs condition were instructed to listen to the audio recording of the intervention three times before Session 3.

Session 3: At the beginning, sleep quality and duration were collected again. This was followed by the intrusion questionnaire. Subsequently, the free recall was performed for the second time and the cued recall for the first time. In addition, participants were asked whether they had talked to others about the TSST or written a diary, and whether they had ever experienced a similar event before.

Session 4: The last survey took place online, and participants were sent a link to complete it at home. At the beginning, the intrusion questionnaire was presented for the fourth time. Following this, the cued recall was administered for the second time. Additionally, participants were asked about the supposed intention of the study. By means of a debriefing at the end of this session, participants were informed about the purpose and objectives of the study, and it was explained that they had not actually been recorded on video during the TSST.

The experimenter for Session 1 and Session 3 and participants were blind to the intervention condition. Session 2 was conducted by a clinical psychologist.

### Statistical analyses

Data analyses were conducted using SPSS (IBM SPSS Statistics, version 24). All hypotheses were tested two-sided with a significance level of *α* = 0.05.

Condition differences regarding sociodemographics and control variables were examined with independent *t*-tests and chi-square tests.

We calculated 2 (Condition) × 2 (Time) ANOVAs to assess the effect of the TSST and the intervention, respectively, on participants’ moods.

Lastly, 2 (Condition) × 2 (Time) ANOVAs were used to assess the effect of the interventions on participants’ free recall and cued recall.

The assumptions for parametric tests were checked. When testing differences of independent groups, following the recommendations of Bühner and Ziegler (2017), a *t*-test was still used in case of violation of the normal distribution assumption, a *t*-test for heterogeneous variances was used in case of variance heterogeneity, and the nonparametric *U*-test would have been used only in case of violation of one of the conditions in combination with an excess probability close to the significance threshold (0.04 < *p* < 0.06) which was not the case in our data. As ANOVAs are considered robust to violations of the normal distribution assumptions (Harwell et al., 1992) and are less sensitive to variance heterogeneity (Field, 2013) when the groups are approximately equal in size, mixed ANOVAs were used even when the assumptions of normality and variance homogeneity were violated.

## Results

### Baseline differences in control variables

The two conditions did not differ regarding any of the sociodemographic or control variables (see Table 1).

### Manipulation check

#### Trier Social Stress Test

Descriptive statistics of the PANAS pre-TSST and post-TSST are presented in Table 2. To check whether the TSST was experienced as stressful for participants, two mixed 2 (Condition: ImRs vs. NIC) × 2 (Time: pre-TSST vs. post-TSST) ANOVAs were performed. Concerning negative affect, there was a main effect of time showing that negative affect was significantly higher post-TSST than pre-TSST, *F*(1, 96) = 77.20, *p* < 0.001, $\eta_{{\text{p}}}^{2}$ = 0.45. There was neither a main effect of the Condition, *F*(1, 96) = 1.84, *p* = 0.18, $\eta_{{\text{p}}}^{2}$ = 0.02, nor a Condition × Time interaction, *F*(1, 96) = 0.05, *p* = 0.82, $\eta_{{\text{p}}}^{2}$ = 0.001. In contrast, positive affect did not change over time, *F*(1, 96) = 1.70, *p* = 0.20, $\eta_{{\text{p}}}^{2}$ = 0.02. There was also no main effect of Condition, *F*(1, 96) = 0.13, *p* = 0.72, $\eta_{{\text{p}}}^{2}$ = 0.001 and no interaction effect between Condition and Time, *F*(1, 96) = 1.70, *p* = 0.20, $\eta_{{\text{p}}}^{2}$ = 0.02.

**Table 2:** Means (*M*), standard deviations (*SD*) and confidence intervals (CI) of positive and negative affect (PANAS) pre- and post-TSST for both conditions

PANAS	Condition				
	ImRs (*n* = 49)		NIC (*n* = 49)		
	*M* (*SD*)	95% CI	*M* (*SD*)	95% CI	
Positive affect					
Pre-TSST	30.10 (6.28)	[28.30; 31.90]	30.49 (6.85)	[28.52; 32.46]	
Post-TSST	30.38 (8.46)	[26.79; 30.81]	28.80 (7.06)	[27.98; 32.78]	
Negative affect					
Pre-TSST	12.94 (4.25)	[11.72; 14.16]	11.67 (2.31)	[11.01; 12.34]	
Post-TSST	18.24 (7.31)	[16.14; 20.26]	17.26 (5.27)	[15.88; 18.88]	

*ImRs* intervention condition, *NIC* no-intervention control condition, *PANAS* Positive and Negative Affect Schedule, *TSST* Trier Social Stress Test

To test whether the TSST triggered intrusions, descriptive statistics of the intrusion measures were calculated and are presented in Table 3.

**Table 3:** Means (*M*), standard deviations (*SD*) and confidence intervals (CI) of intrusion measures at sessions 1, 2, 3 and 4

Intrusion measures	Total (*n* = 93)	
	*M* (*SD*)	95% CI
Number of intrusions		
Session 1	3.09 (9.72)	[1.08; 5.09]
Session 2	2.19 (3.12)	[1.55; 2.84]
Session 3	0.86 (1.52)	[0.55; 1.17]
Session 4	1.24 (2.40)	[0.74; 1.73]
Percent of time with intrusions		
Session 1	20.22 (24.49)	[15.17; 25.26]
Session 2	9.25 (12.87)	[6.60; 11.90]
Session 3	4.41 (8.40)	[2.68; 6.14]
Session 4	5.05 (11.86)	[2.61; 7.49]
Vividness of intrusions		
Session 1	23.12 (27.86)	[17.38; 28.86]
Session 2	15.81 (20.13)	[11.66; 19.95]
Session 3	9.78 (19.34)	[5.80; 13.77]
Session 4	8.92 (15.64)	[5.70; 12.14]
Distress of intrusions		
Session 1	19.14 (25.86)	[13.81; 24.47]
Session 2	11.83 (17.75)	[8.17; 15.48]
Session 3	5.70 (13.78)	[2.86; 8.54]
Session 4	6.88 (16.55)	[3.47; 10.29]
Controllability of intrusions		
Session 1	40.43 (39.56)	[32.28; 48.58]
Session 2	45.27 (40.96)	[36.83; 53.70]
Session 3	23.87 (37.10)	[16.23; 31.51]
Session 4	26.77 (38.11)	[18.93; 34.62]

*Session 2*: 2 days after Session 1, *Session 3*: 1 week after Session 2, *Session 4*: 3 months after Session 3

#### Intervention

To check whether the intervention had an influence on participants’ positive and negative affect, two mixed 2 (ImRs vs. NIC) × 2 (pre-intervention vs. post- intervention) ANOVAs were performed. Descriptive statistics are presented in Table 4.

**Table 4:** Means (*M*), standard deviations (*SD*) and confidence intervals (CI) of positive and negative affect (PANAS) pre- and post-intervention for both conditions

PANAS	Condition				
	ImRs (*n* = 49)		NIC (*n* = 50)		
	*M* (*SD*)	95% CI	*M* (*SD*)	95% CI	
Positive affect					
Pre-intervention	27.71 (6.22)	[25.93; 29.50]	27.26 (6.82)	[25.32; 29.20]	
Post-intervention	32.53 (7.81)	[30.29; 34.77]	27.36 (6.85)	[25.41; 29.31]	
Negative affect					
Pre-intervention	13.04 (3.97)	[11.90; 14.18]	11.96 (2.08)	[11.36; 12.55]	
﻿Post-intervention	12.88 (3.78)	[11.79; 13.96]	12.14 (2.01)	[11.57; 12.71]	

*ImRs* intervention condition, *NIC* no-intervention control condition, *PANAS* Positive and Negative Affect Schedule

Looking at the positive affect pre- and post-intervention, the ANOVA showed a main effect of Time, *F*(1, 97) = 24.78, *p* < 0.001, $\eta_{{\text{p}}}^{2}$ = 0.20 and a main effect of Condition, *F*(1, 97) = 4.65, *p* = 0.03, $\eta_{{\text{p}}}^{2}$ = 0.05. These were qualified by an interaction effect of Condition and Time, *F*(1, 97) = 22.34, *p* < 0.001, $\eta_{{\text{p}}}^{2}$ = 0.19. As shown in Table 4, positive affect increased after ImRs, *t*(48) = -5.86, *p* < 0.001, *d* = 0.84, but not after NIC, *t*(49) = -0.18, *p* = 0.86, *d* = 0.02.

For negative affect, the ANOVA yielded no main effect of Condition, *F*(1, 97) = 2.86, *p* = 0.09, $\eta_{{\text{p}}}^{2}$ = 0.03, no main effect of Time, *F*(1, 97) = 0.001, *p* = 0.98, $\eta_{{\text{p}}}^{2}$ = 0.00, and no interaction effect between Condition and Time, *F*(1, 97) = 0.30, *p* = 0.58, $\eta_{{\text{p}}}^{2}$ = 0.003.

### Free recall

Descriptive statistics for correct and incorrect details are presented in Table 5. The effect of the intervention on voluntary memory measured by free recall was investigated by two mixed 2 (ImRs vs. NIC) × 2 (Session 2 vs. Session 3) ANOVAs for the number of correct and incorrect details. Additionally, to control whether the length of the free recall differed between conditions, a further ANOVA with the same factors was run for the total number of reported details.

**Table 5:** Means (*M*), standard deviations (*SD*) and confidence intervals (CI) of the results for free recall at sessions 2 and 3 and cued recall for sessions 3 and 4

Free recall	Condition			
	ImRs (*n* = 50)		NIC (*n* = 49)	
	*M* (*SD*)	95% CI	*M* (*SD*)	95% CI
Number of correct details				
Session 2	34.00 (12.49)	[30.45; 37.55]	33.04 (12.39)	[29.48; 36.60]
Session 3	40.26 (11.41)	[37.02; 43.50]	33.96 (12.92)	[30.25; 37.67]
Number of incorrect details				
Session 2	3.02 (2.44)	[2.33; 3.71]	3.24 (2.98)	[2.39; 4.10]
Session 3	3.14 (2.72)	[2.05; 3.79]	2.92 (3.03)	[2.37; 3.91]
Total number of details				
Session 2	123.84 (54.38)	[107.91; 139.77]	112.51 (59.11)	[96.41; 128.61]
Session 3	132.10 (45.58)	[116.99; 147.21]	108.84 (61.13)	[93.57; 124.10]

	ImRs (*n* = 49)		NIC (*n* = 47)	
Cued recall	*M* (*SD*)	95% CI	*M* (*SD*)	95% CI
Correct answers				
Session 3	30.90 (5.14)	[29.42; 32.37]	30.60 (5.83)	[28.88; 32.31]
Session 4	26.78 (5.61)	[25.16; 28.39]	26.87 (7.22)	[24.75; 28.99]
Incorrect answers				
Session 3	7.71 (3.40)	[6.74; 8.69]	8.17 (3.55)	[7.13; 9.21]
Session 4	8.82 (5.03)	[7.37; 10.26]	9.02 (4.90)	[7.58; 10;46]
“*I do not know*”				
Session 3	6.86 (3.13)	[5.96; 7.76]	6.38 (3.67)	[5.31; 7.46]
Session 4	9.65 (4.39)	[8.39; 10.91]	9.21 (6.50)	[7.31; 11.12]

*ImRs* intervention condition, *NIC* no-intervention control condition*, Session 2*: 2 days after Session 1, *Session 3*: 1 week after Session 2, *Session 4*: 3 months after Session 3

Looking at the number of correct details, there was a significant main effect of Time, *F*(1, 97) = 23.99 *p* < 0.001, $\eta_{{\text{p}}}^{2}$ = 0.20, but no significant main effect of Condition, *F*(1, 97) = 2.36, *p* = 0.13, $\eta_{{\text{p}}}^{2}$ = 0.02. The Time effect was qualified, however, by a significant Time $\times$ Condition interaction, *F*(1, 97) = 13.28, *p* < 0.001, $\eta_{{\text{p}}}^{2}$ = 0.12. Whereas the number of correctly remembered details increased following ImRs, *t*(49) = -5.96, *p* < 0.001, *d* = 0.84, there was no significant change in the number of correctly remembered details in NIC over time, *t*(48) = -0.90, *p* = 0.37, *d* = 0.13.

Looking at incorrect details, the ANOVA yielded neither a significant main effect of Time, *F*(1, 97) = 0.17, *p* = 0.68, $\eta_{{\text{p}}}^{2}$ = 0.002, nor a significant main effect of Condition, *F*(1, 97) = 0.00, *p* > 0.99, $\eta_{{\text{p}}}^{2}$ = 0.00, nor a Time $\times$ Condition interaction, *F*(1, 97) = 0.78, *p* = 0.38, $\eta_{{\text{p}}}^{2}$ = 0.01.

To control for the total verbal output, we also compared the total details of Sessions 2 and 3. Descriptive statistics are also presented in Table 5. The ANOVA yielded neither a significant main effect of Time, *F*(1, 97) = 0.58, *p* = 0.45, $\eta_{{\text{p}}}^{2}$ = 0.01, nor a significant main effect of Condition, *F*(1, 97) = 2.61, *p* = 0.11, $\eta_{{\text{p}}}^{2}$ = 0.03, nor a Time $\times$ Condition interaction, *F*(1, 97) = 3.90, *p* = 0.05, $\eta_{{\text{p}}}^{2}$ = 0.04.

### Cued recall

Descriptive results for the cued recall task are shown in Table 5. The effect of the intervention on voluntary memory measured by a cued recall test was examined by three mixed 2 (ImRs vs. NIC) × 2 (Session 3 vs. Session 4) ANOVAs for the sum scores for correct, incorrect and “*I do not know*” answers.

Looking at the number of correctly remembered features in the cued recall, we found a significant main effect of Time, indicating that the number of correctly remembered details decreased from Session 3 to Session 4 in both conditions, *F*(1, 94) = 73.11, *p* < 0.001, $\eta_{{\text{p}}}^{2}$ = 0.44. There was neither a significant main effect of the Condition, *F*(1, 94) = 0.01, *p* = 0.93, $\eta_{{\text{p}}}^{2}$ = 0.00, nor a significant Time $\times$ Condition interaction effect, *F*(1, 94) = 0.19, *p* = 0.67, $\eta_{{\text{p}}}^{2}$ = 0.002.

The ANOVA for the number of incorrect details also showed a significant effect of Time, *F*(1, 94) = 5.24, *p* = 0.02, $\eta_{{\text{p}}}^{2}$ = 0.05, indicating that the number of incorrect remembered details significantly increased over time in both conditions. Again, the main effect of Condition, *F*(1, 94) = 0.19, *p* = 0.67, $\eta_{{\text{p}}}^{2}$ = 0.002, and the Time $\times$ Condition interaction, *F*(1, 94) = 0.09, *p* = 0.77, $\eta_{{\text{p}}}^{2}$ = 0.001, were not significant.

Similarly, the number “*I do not know*”-answers increased between Session 3 and 4 for both conditions, *F*(1, 94) = 41.19, *p* < 0.001, $\eta_{{\text{p}}}^{2}$ = 0.31. Again, the effect of Condition, *F*(1, 94) = 0.31, *p* = 0.58, $\eta_{{\text{p}}}^{2}$ = 0.003, and the Time $\times$ Condition interaction, *F*(1, 94) = 0.001, *p* = 0.97, $\eta_{{\text{p}}}^{2}$ = 0.00, were not significant.

## Discussion

This study investigated the effect of ImRs on voluntary memory of an aversive autobiographical event in a healthy sample. Given the scarcity of studies in this area, the findings contribute to the debate about whether trauma-focused imagery-based interventions diminish memory accuracy of autobiographical (traumatic) events.

Contrary to expectations, ImRs did not increase the number of incorrect details reported in the free recall. However, we observed that the number of correct details reported in free recall increased in ImRs compared to the number reported in NIC. Hence, free recall findings did not support the assumption that ImRs deteriorates autobiographical memory, but instead suggest that the validity of voluntary autobiographic memory might even *improve* as a result of ImRs.

Results of the cued recall did not show any differential effects between conditions. However, participants’ memory performance decreased over time in both conditions (i.e., increase in incorrect answers, decrease in correct answers). This was paralleled by a decrease in cued recall performance (i.e., higher number of features not remembered) in both conditions over the 3-month follow-up period. These findings of memory deterioration over time across tasks are most likely due to normal forgetting processes (MacLeod, 2002).

Although some researchers (Brainerd & Reyna, 2005; Volbert & Steller, 2014) have claimed that most guided imagery-based techniques are similar to false memory procedures, taken together, we did not find evidence that ImRs distorts voluntary memory. According to the Source-Monitoring Framework (Johnson, 2006; Johnson et al., 1993), imagined events may be mistaken for actual events if these events share some similarity with each other. Hence, if individuals are guided by ImRs to rescript imagined scenarios that are rich in perceptual details and emotional valence, it is conceivable that this may induce the belief that the imagined scenarios have actually taken place. However, our study does not support this assumption. Foley et al. (2006) assume that the extent of memory distortions depends very much on the characteristics of the instructions used for the imagery script. Their results suggest that it is mainly the source of the imagery script (oneself or another person) that affects reports of false memories. They observed a reduction in the error rate when participants generated the content of the imagery themselves. This was mainly the case in the intervention group of our study and may explain our results. If this result can be replicated, careful attention should be paid to the extent to which participants control their own imagery scripts in ImRs. In clinical practice, it is occasionally the therapist who initially performs rescripting, especially in childhood trauma and in more severe patients. According to the results and considerations of Foley et al. (2006), this might be critical. Our results may provide preliminary evidence that it is less critical to let the patients themselves be the authors of the rescripting in the first place. Moreover, transferring the results of Karanian et al. (2020), who warned individuals about the threat of misinformation by a simple warning and thus were able to reduce misinformation effects, it seems crucial to inform patients about the rational and the procedure of ImRs in detail before starting the rescripting process (i.e., to make the difference between reality and the script explicit and transparent).

The assumption that source errors are more likely when imagery is generated unintentionally than when it is generated intentionally is consistent with the view of the Source-Monitoring Framework. Enhanced cognitive operations associated with a memory can make participants aware that the change was internally generated and thus facilitate discrimination (Henkel & Carbuto, 2008). Since ImRs involves making the integration of new information into memory salient, the active generation of script changes by participants may also make them more likely to encode and remember the cognitive operations of the ImRs procedure, which may prevent the memory from distorting. This also fits with the idea that ImRs rather builds up new memory representations than distorting existing ones. According to the ideas on working mechanisms (Arntz, 2012; Arntz & Weertman, 1999) ImRs is supposed to change the meaning of the fear memory (i.e., reevaluates the UCS representation) by forming a new less-distressing memory representation and is not supposed to replace or erase the factual details of the original memory representation. Following the retrieval competition hypothesis (Brewin, 2006), it could also be assumed that ImRs does not directly alter symptomatic semantic or episodic memories, but creates competing representations that integrate new positive elements into existing negative material (Brewin et al., 2010). If this process is successful, a sufficiently positive memory representation that neutralizes existing negative emotions and is sufficiently memorable wins the retrieval competition with the original negative and stressful representation (Brewin et al., 2009).

Moreover, our findings indicate that ImRs might even lead to an improved memory. The revised Dual Representation Theory (Brewin et al., 2010) provides a possible explanation for this finding. It states that he following two different types of memory representation are encoded during a traumatic event: (1) a sensory and emotion-laden representation of the traumatic event (S-rep), and (2) a more abstract structural representation that only includes a subset of the sensory input along with contextual information (C-rep). While in healthy memory C-rep is voluntarily accessible and tightly associated with S-rep, i.e., S-rep is retrieved via the associated C-rep, in trauma memory C-rep is either encoded only weakly or without the associated S-rep. Hence, the S-rep often is directly and involuntarily activated following trauma. ImRs, which includes retrieval of both the contextualized representations of the traumatic memory and the sensory-bound representations, might allow all relevant material in the sensory-bound representation to be fully contextualized by assigning it to a new and more elaborated, contextualized representation. Hence, it strengthens the association between these two types of memory representation, which might in turn support consolidation and improve the verbal accessibility of declarative memory (reflected by the increased number of correct memory details found in the current study). However, as an active control was missing in our study, we cannot rule out the possibility that improved memory following ImRs was caused by the repeated listening to the recorded rehearsal of the memory activation part, which was not part of the NIC. This alternative and rather simple explanation is also indicated by a trend in the Time $\times$ Condition interaction towards an increase of the total number of details in free recall. This is in line with the results of Romano et al. (2020), who investigated the effects of ImRs on memory performance in social anxiety disorder. ImRs was compared with Imaginal Exposure (IE) and supportive counselling. Depending on the condition, the content of autobiographical memory representations changed in different ways: ImRs only promoted the increase of positive and neutral memory details, while IE promoted the increase of both positive, neutral and negative memory details, and supportive counselling did not induce any changes. The authors assume that the interventions each facilitated an increase in focused content, i.e. positive/neutral details in ImRs and both positive/neutral and negative details in IE, while there was no change in supportive counselling. Our data could also be explained by findings from the Cognitive Interview literature. Nori et al. (2014) found that after interviewing with imagery, more correct information was remembered than after interviewing without imagery. In addition, as in our case, there was no increase in confabulations and false information following imagery. Nori et al. assume that the ability to remember a stimulus depends on the similarity between the way the stimulus is processed during encoding and the way it is processed during remembering. Even though in our study all participants had some imagery at least in free recall, the higher dose in ImRs may have a comparable effect. Accordingly, repeated imagery and associated reencoding (especially of sensory information) could facilitate a recall using imagery, whereas it would not affect a pure verbal recall. This is in line with our results, showing that ImRs improved the free recall (in which participants were explicitly asked to imagine the TSST experience) whereas we found no group differences in the cued recall (in which participants were not instructed to imagine their experience). Since listening to audio recordings of ImRs sessions is not standard in many applications (at least not in all RCTs published to date), our results may also not be generalizable to all ImRs protocols. However, because of the higher dose due to listening, it is more likely that the effects of ImRs on voluntary memory are overestimated rather than underestimated.

The fact that ImRs did not deteriorate voluntary memory is in line with earlier studies looking at the effect of ImRs on voluntary memory in secondary analyses (Hagenaars & Arntz, 2012; Siegesleitner et al., 2019), and extends these results from studies using the trauma film paradigm to aversive *autobiographical* memories. Furthermore, this is the first study including a free recall task, which arguably shows a higher external validity than cued recall, and a three-month follow-up. In sum, results from the current study and the two earlier studies investigating the effects of ImRs on voluntary memory provide evidence that ImRs does not necessarily deteriorate (short- and long-term) voluntary memory. Preliminary results suggest that ImRs might even improve voluntary memory, but the latter finding is somewhat less consistent across studies.

The current findings compete with the wider basic memory literature showing that imagination can lead to distortions of voluntary memory (Garry et al., 1996; Goff & Roediger, 1998; Nash et al., 2009; Thomas & Loftus, 2002). Thus, although imagery-based interventions *can* lead to memory distortions, currently available evidence does not suggest that this *regularly* happens when applying a low dosage (only one session) of imagery-based intervention, even if these include imagining the experienced event in a counterfactual way. This raises the question of what moderates these differential effects of imagination on voluntary memory.

On the one hand, the goals, rationale, and exact procedures of imagery-based interventions could be of relevance. The aforementioned studies (e.g., Garry et al., 1996) looking at false memory effects explicitly asked for additional information or suggested a context for the imagination by using imagination inflation (i.e., imagining events that supposedly took place) or memory implantation (i.e., false testimony of family members or faked photos) (Brewin & Andrews, 2017). In contrast, the instructions used in state-of-the-art trauma-focused treatment make the purpose of the intervention transparent by simply asking patients to imagine the event in a first step and to integrate their own helpful perspectives in a second step to make the traumatic event less emotionally distressing (Arntz & Weertman, 1999).

On the other hand, it may be important how declarative change is measured. Previous studies (Garry et al., 1996; Goff & Roediger, 1998; Nash et al., 2009; Thomas & Loftus, 2002) did not assess changes of relevant details and facts about people, time, places, or actions, but instead assessed confidence regarding whether a previously performed and/or imagined action or event had actually taken place. Interestingly, empirical research suggests that believing that an event occurred and recollecting this event is not associated (Hart & Schooler, 2006; Pezdek et al., 2006), and may be even more dissociated than previously assumed (Roediger et al., 2012; Scoboria et al., 2014). Thus, subjective confidence is not qualified to predict memory accuracy.

Some limitations of the current study have to be kept in mind. First, TSST exposure is not comparable with experiencing a genuine trauma and is rather a mild manipulation, as indicated by not very high negative affect scores and stable positive affect scores throughout TSST. Future research could modify the TSST to further intensify negative affectivity and reduce positive affectivity. However, TSST still led to large increases in negative affect and triggered as many intrusions within a week as various trauma films did in previous studies (Arnaudova & Hagenaars, 2017). Furthermore, analogue designs inducing a negative to-be-remembered event are necessary to assess the accuracy of an episodic memory, which was of great importance in our study and is not possible for naturalistic events that are beyond experimental control.

Nevertheless, the generalizability of our results to a PTSD patient sample are limited (Brewin, 2007). On the one hand, strong emotions in PTSD patients might impair source monitoring (Johnson, 2006), making it more difficult to monitor reality and distinguish between imagined and actually experienced features. On the other hand, there is evidence that increased emotional arousal narrows attention to the central aspects of the event, though possibly at the expense of peripheral details (Kaplan et al., 2016). However, this suggests that PTSD patients are particularly good at remembering the relevant details anyway. It remains unclear how memory distortions (a common symptom of PTSD) influence our results. Evidence from a study by Bedard-Gilligan et al. (2017) examining the effect of imagery-based exposure therapy on voluntary memory in PTSD patients showed that the number of sensory details reported increased while memory quality (i.e. fragmentation) did not change between pre- and post-treatment. Furthermore, both the revised Dual Representation Theory (Brewin et al., 2010) and the cognitive model of PTSD (Ehlers & Clark, 2000) assume that traumatic memories are encoded differently as compared to non-traumatic memories. This also limits the generalizability of our results to PTSD samples and has to be further investigated in future research. Second, an active control was missing in our study. Memory for the event may have been bolstered simply by discussing the event for 15 min – even without any ImRs. Therefore, as a next step, future studies should also include another control condition that discusses the event without using any imagery. Third, we used individualized ImRs scripts to increase the external validity of the intervention. However, this may have caused a greater variance with respect to the effects ImRs had on participants’ voluntary memory, e.g., depending on which part of the TSST experience was rescripted. Increased variance may have made it more difficult to identify intervention effects on memory and could be controlled in future studies. In addition, like previous studies (Hagenaars et al., 2008; Siegesleitner et al., 2019), we also applied only a single session of ImRs. In studies with clinical samples, the average number of sessions was 4.5, with a range of 1–16 sessions (Morina et al., 2017). Even though we were at the lower end of the dosage spectrum compared to the use of ImRs in psychological treatment, a single session has also been shown to be effective (Grunert et al., 2007). Another limitation is that ImRs was presented relatively soon after the TSST. In clinical practice, there might be months or even years between a traumatic experience and the application of ImRs. Pansky et al. (2011) assume that memory representations consist of features that are interconnected to some degree, however, differ in the number of features encoded and in the strength of the connections between them, both of which determines their recall ability. Based on the Source-Monitoring Framework (Johnson, 2006; Johnson et al., 1993), they assume that the connections linking features become weaker over time, so that some of the features are lost and lead to partial degradation of the original memory trace, potentially facilitating distortions. On the other hand, traumatic memories are considered to be particularly good to remember and appear to be recalled more reliably over time than non-traumatic emotional experiences (Peace & Porter, 2004). According to Goodman et al. (2017), even later adult memories of stressful to threatening childhood experiences are more accurate the more traumatic the event and the more traumatic its effects (e.g., more PTSD symptoms) were. In summary, a lack of personal relevance (as is often the case in basic studies on false memories) seems to be more crucial for susceptibility to distortion than time between event and intervention. However, further research is needed to clarify this definitively.

In sum, this study provides important novel evidence regarding the ongoing debate about whether imagery-based interventions might reduce memory accuracy of distressing events. Contrary to commonly held assumptions, no memory deterioration caused by ImRs was observed. This may weaken the position that after imagery-based interventions, survivors’ accounts of their traumatic experiences cannot be deemed credible in the legal context. Importantly, we even observed an improvement of voluntary memory following ImRs. Future theoretical development and empirical research is needed to specify the circumstances under which trauma-focused treatment does or does not influence voluntary memory. This will help both victims and therapists to balance therapeutic and judicial concerns.

## Supplementary Information

Below is the link to the electronic supplementary material.

Supplementary file1 (DOCX 32 KB)

## Data Availability

Anonymized data have been made publicly available at the https://osf.io/ and can be accessed at https://osf.io/x2c5u/?view_only=d7638bd261c44b70bbb8e6886a1a7859

## Footnotes

1 *Declarative voluntary* memory is abbreviated as *voluntary* memory in the following.

2 Additionally, participants completed the Cognitive Emotion Regulation Questionnaire (CERQ; Loch et al., 2011), Difficulties in Emotion Regulation Scale (DERS; Ehring et al., 2013), Emotion Regulation Questionnaire (ERQ; Abler & Kessler, 2009), Stress Appraisal Measure (SAM; Delahaye et al., 2015), Multidimensional Mood Questionnaire (MMQ; Steyer et al., 1997), and the Heidelberg Form for Emotion Regulation Strategies (HFERST; Izadpanah et al., 2019) in Session 1. HFERST was repeated in Session 2, Session 3 and Session 4. SAM and MMQ were repeated in Sessions 3 and 4. However, these questionnaires are independent of the current research question and the results will be reported elsewhere. Therefore, for reasons of clarity, these measures will not be processed further in the present manuscript.
