# Supplementary material for: The dilemma of trauma-focused therapy: effects of imagery rescripting on voluntary memory
Source: Psychol Res. 2022 Nov 5;87(5):1616–31. doi: 10.1007/s00426-022-01746-z (PMC10227147; doi:10.1007/s00426-022-01746-z)
Supplement: Supplementary file 1 — Supplementary file1 (DOCX 32 KB) [file 426_2022_1746_MOESM1_ESM.docx]

**Supplementary Material**

**Supplementary Material A**

***Free recall (memory reactivation) – instructions (English translation)***

"*The following will, once again, be about the event that took place two days ago. I will ask you to talk about it and audio record this sequence of your narration.*

*Can you remember the situation from the day before yesterday? You made a presentation to a committee the day before yesterday and performed other tasks. In the next step, we will once again go over the experience you had the day before yesterday in your imagination - that is, in your mind's eye. I’ll ask you to retell your experience as vividly as possible right away. In order to see this new course of the experience as vividly as possible in front of you and to experience it with all the bodily sensations, feelings that arise, and with as many details as possible, you should close your eyes and speak in the first person and in the present tense. It is also important to pay attention to all sensory channels while doing this. So, let’s discuss what you see, what you hear, what you smell, what you feel, etc.*

*I would like to demonstrate this with a small example: For example, if I want to experience my breakfast from this morning as vividly as possible, I do it in the following way.* [experimenter closes eyes (be sure to do this!), please recite next part by heart, participants will feel less weird when they keep their eyes closed themselves]: *'I'm in the kitchen now, thinking about what I want to eat. It's eight o'clock in the morning. I'm still in my pajamas and a little tired, but I'm also looking forward to the day. I go to the refrigerator and open it with my left hand. The air coming out of the fridge is cold and the fridge makes a whirring sound. It smells like fresh coffee. My eyes fall on a jam jar and I realize I'm in the mood for a jam sandwich. I take the jar out of the fridge and close the door again...'* [open your eyes]. *And so on...*

*I ask you to imagine the course of your experience from the day before yesterday as vividly as possible in this way as well. So, in the present tense, as if you were there at this moment, and from your perspective, tell me what you see, feel, hear, etc. in the moment.*

*Please describe the event as detailed and as accurately as possible. Please start at the point where the committee enters the room and end at the point where the committee leaves the room. It is important that you describe everything you can remember in such a way that a listener unfamiliar with the event can imagine the scene as accurately as possible. I will not interrupt you while you are speaking but will simply listen until you decide to finish your narrative. If you forget to narrate in the present tense, I might briefly correct you. Please don’t be irritated by this and please continue describing the event in the present tense. While doing so, please remember to actually describe the event and all the details as completely and accurately as possible. Please report what was done and said and what you saw. Now make yourself comfortable in the chair and close your eyes. You may begin.*"

[Use audio device for free recall, start audio recording, speak participant code on tape]

[Participant reports recollection; min. 3 min.]

[If respondent falters] "*And what happens next?*"

**ATTENTION:** Respondent should always report in the first person (correct: "*Please remember to describe the event in the first person*") and in the present tense (gently correct: repeat what was said in the present tense) (e.g., "*So the commission is taking a seat at the table right now. What happens now?*")

[If the participant digresses into his or her report from the TSST and reports events from Session 1 that happened before or after the TSST]: "*For our study, it is important that we focus on the presentation from the day before yesterday. That's why I’d like you to come back to the situation with the committee in terms of content. You said* [insert here the last aspect of the scene described by the participant]*. What's next?*"

[Have participant narrate until the time the commission leaves the room again. If participant stops before this then ask]: "*Does this conclude the situation? Please continue telling the story until the commission leaves the room*."

**Supplementary Material B**

***Imagery Rescripting – instructions (English translation)***

"*In the situation with the committee, what was the most uncomfortable moment for you? Can you describe this very briefly*?" [note hotspot].

"*In the next step, we will go through the experience again in your imagination. So right now, I'm going to ask you to imagine the situation again as vividly as possible and describe to me what you’re experiencing. Please describe your experience again in the first person and in the present tense, as if it were happening right now. It is important to pay attention to all sensory channels. So: What do I see? What do I hear? What do I smell? What do I taste? What do I feel? Let the image of the beginning of the scene arise before your inner eyes and then describe the course of the scene. The point is not that I understand everything exactly, but that you imagine the scene vividly and share what you experience. In a moment, I will help you to create the image of the beginning of the scene in your mind's eye, and then you will first describe the beginning of the scene again. At a certain point we will change the script of the scene – I will let you know at which point and how exactly we will do this. I will accompany you in the imagination exercise by asking you questions from time to time. Throughout the exercise, please keep your eyes closed. The exercise will take about 10-20 minutes. I will then signal you when the exercise is over, and you can open your eyes again. Do you have any questions before we start?*" [Answer questions, but nothing about the purpose of the study or imagery rescripting]

"*All right, I will now help you recall the experience again as vividly as possible. For this, I will first repeat what happened. I ask you then to imagine it as vividly and in as much detail as possible. You do not need to do anything else yet. Now make yourself comfortable in the chair and close your eyes.*" [display on the audio recording device serves as a stopwatch, note the duration of the recording after you have finished]

[use second audio device that participant takes home; start audio recording]. "*You have just prepared for your presentation and are sitting in the chair across from the table. ("Do you see this in front of you?") The investigator is just getting the application committee. You are sitting alone in the examination room. The door opens. The two members of the application committee come in. They sit down at the table. There are many documents on the table in front of them. Directly across from you is a video camera. One of the committee members turns on the video camera and says, ‘Start recording’ and the participant code. The committee members look at you. One of them tells you that you can start the presentation. What happens now?*"

[If participant falters] "*And what happens next?*", "*What's happening now?*"

**NOTA BENE:** Participant should always report in the first person (correct: "*Please remember to describe things using the first person*", and in the present tense (correct: repeat what was said in the present tense) (e.g., "*So the commission is taking a seat at the table right now. Where do we go from here?*").

[Questions to explore]: “*Where are you?, What are you doing?, Who else is present?, What are they doing?, What can you see?, What can you hear?, Can you smell or taste anything?, What are you thinking?, How do you feel?, What physical sensations do you have? (have them describe things in detail), Where are you feeling this?, How does it feel?*”

[Let the participant tell until they reach the hotspot, then]: "*You may now change the script of the scene to make it less stressful. The change can be something that could really happen or something that would be impossible in reality. Anything is possible, we just can't undo the event. It is important that you change the course of the scene so that it is less stressful for you. So, you are now* [insert hotspot point of view]. *What would you like to do or say now to change the situation? Can you visualize yourself doing that now? What happens next?"*

"*Please imagine this as vividly as you can, as if this is really happening right now! And please tell me exactly what is happening.*"

[Questions to deepen or if very global, e.g., "*I want everything to be fine*."]: "*What might that look like specifically if...?, Can you imagine exactly how you would do this?, What do you need to do this?, What exactly are you doing?, What happens now?, How does the commission respond?, What exactly does* [the helper] say/do*?, Imagine exactly how he says/does this, Is there anything else you need* [the helper] *to do or anything you want* [the helper] *to do?, How does it feel when you ...?, What do you think?, Is there anything else you need?, Is there anything else you would like to change?"*

*"Does this feel completely good to you now, or is there anything else you would like to change about the story so that it feels completely good to you?"* [Repeat until participant is satisfied. Ideally about 10-20 minutes].

"*Well, if this feels completely good to you now, then you can let this picture sink in for a moment. And when you're ready, we'll end the imagination session with that, and you can open your eyes again.*"

[End Audio Recording]

[Read duration of rescripting from audio recording device. Log time of rescripting in checklist.]

**Supplementary Material C**

***Free recall – summary rating manual***

**Internal and external details based on Levine et al. (2002)**

***Categories of internal details.*** *Event*: Activities and actions of one person and reactions of others (jury) (*subject + verb*) are evaluated as an event: actions of the participants (presentation, arithmetic task, singing, also the attempt at a visible behavior) (e.g., "*I notice*", "*I wait*", "*I lean*") and actions and reactions by the jury (e.g., camera on, comments). In addition, clothing, physical appearance and any adjective that describes a person in more detail will be counted as an event when first mentioned (e.g., “*lady with the red dress and the brown, long, curly hair*”).

*Place*: As a place, all location information (room, building, cross on the floor) and prepositions (right, opposite, I stand in front of the table etc.) are evaluated. Each preposition is scored separately with one point.

*Time:* All temporal adverbs (then, after, next, etc.), information about the order (first, last, at the end, at the beginning, as the second, etc.) and the duration (5 min, finished with speech before time runs out), as well as the time, day or date, are coded as time.

*Perception*: All auditory, tactile (including pain), visual, and olfactory details (what the participants see, hear, etc.), as well as object characteristics (shape and color of e.g., the blackboard), additional information on body position / posture (“*I stand upright/bent*”) and indirect speech (“*the commission says I should speak louder”*) are considered perception. Each object property/descriptive adjective (white, large, square board) is scored separately with one point. (Adjectives describing persons are coded as an event.) Each statement / detail in indirect speech (in accordance with TSST protocol) is scored separately with one point.

*Emotion / cognition*: All expressions concerning the emotional state, thoughts, evaluations, or expectations are evaluated as emotion or cognition (e.g., "*what is actually not a problem*", "*I find it difficult*", "*the commission seems very professional*", "*even if it doesn't work out quite as well with the arithmetic*", "*I am curious about what is coming*", "*I try*"). Indications of emotions are "*I think, feel*". Thoughts that are specified in more detail, or thoughts and feelings that fit together conceptually, are evaluated as one point.

***Categories of external details.*** *Metacognition, semantic knowledge*: Semantic knowledge, self-concept, evaluations in retrospect, additional explanations from the present, and explanations of feelings are evaluated as metacognitions (e.g., "*When I think about it now, ...*", "*but I already knew that from a seminar*", "*I know that I’m not a super singer* ", "*the task is difficult for me because mental arithmetic was so long ago*", *"a blackboard which you can write on*", “*which doesn't even fit to these first, serious tasks*". One indication of metacognition is, for example, the statement "*at this time I have...*").

*Repetitions:* Details that do not supply any new information and whose information was already mentioned analogously are not scored for a second time.

*Others:* Other elements are not considered or evaluated, including filler words (okay, so, exact, etc.), corrections or muddling

**Correctness ratings based on Jack et al. (2014)**

*Correct:* Everything the jury does according to TSST protocol will be rated as correct. In addition, everything that happens within a task, no matter whether the chronology is correct or not, will be rated as correct. If the time is not correct, the time detail will be rated as incorrect.

*Incorrect:* Everything that is wrong according to TSST protocol will be rated as incorrect.

*Possible*: Everything the participant says, perceives and does is considered possible if verification is not possible.

**Supplementary Material D**

***Cued recall – questions and rating manual***

|  | Question | Number of correct answers | Correct answers | Answers not to be scored | Incorrect answers |
| --- | --- | --- | --- | --- | --- |
| Questions about the location | | | | | |
| 1 | Please name all pieces of furniture, furnishings and home accessories that you remember. | 7 | Table, chairs, lamp, picture, plant, curtain, flipchart | Trash can, watering can, colors & number of features, things in room, folders, carpet | All other mentions (things that were not in the room, such as folders) |
| 2 | Was there something on the windowsill? If  yes, what? | 2 | Yes, Watering can | Color & quantity | No, all other mentions |
| 3 | Were there pictures hanging on the wall? If so, how  many? | 2 | Yes, 1 | Content of the pictures | No, all other mentions |
| 4 | Was the room illuminated? If so, which light sources were there? | 3 | Yes, floor lamp, ceiling lamp | Windows, daylight | No, all other mentions |
| 5 | Was there a shelf in the room? If so, what color was it? | 1 | No |  | Yes, all other mentions |
| 6 | What was on the jury table in the room? | 7 | Coffee cups / glasses, water bottle, clipboards, water carafe, stopwatch, pens, papers | Quantity, folders | All other mentions |
| 7 | Was there a trash can in the room? If yes, what color was it? | 2 | Yes, blue | White sticker | No, all other mentions |
| 8 | Were there any folders in the room? If so, how many? | 1 | No |  | Yes, all other mentions |
| 9 | Did the jurors have clipboards?  If so, what color were the jurors' clipboards? | 2 | Yes, blue | Exact blue tone | No, all other mentions |
| 10 | Was there a watering can in the room? If so, what color was the watering can? | 2 | Yes, pink |  | No, all other mentions |
| Questions about the person | | | | | |
| 11 | What color(s) were the jury members' tops? | 2 | Black, red |  | All other mentions |
| 12 | What hairstyle did the juror on your left wear? | 1 | Hair was down |  | All other mentions |
| 13 | What hairstyle did the juror on your right wear? | 1 | Ponytail |  | All other mentions |
| 14 | What color(s) were the jury members' shoes? | 1 | Black |  | All other mentions |
| 15 | Were any of the jurors wearing glasses? If so, what color were they? | 1 | No |  | Yes, all other mentions |
| 16 | Which juror first gave you instructions? | 1 | Left |  | Right |
| 17 | Which juror gave you the last task? | 1 | Left |  | Right |
| 18 | Which juror operated the camera? | 1 | Right |  | Left |
| Questions about the situation | | | | | |
| 19 | What color was the camera? | 1 | Black |  | All other mentions |
| 20 | What color was the marking where you had to stand? | 1 | Brown |  | All other mentions |
| 21 | What was your first task? | 1 | Hold presentation / strengths & weaknesses | More detailed content about the presentation | All other mentions |
| 22 | How long did the presentation last (in minutes)? | 1 | 5 |  | All other mentions |
| 23 | What were you asked to talk about during the presentation? | 1 | Strengths & weaknesses / character traits | Job interview, more details | All other mentions |
| 24 | Were you interrupted during your speech? If so, how often were you interrupted? | 2 | Yes, 5 |  | No |
| 25 | Did you have to perform another task? If so, what was your task? | 2 | Yes, arithmetic task | More specific information about the arithmetic task, singing | No, all other mentions |
| 26 | At the beginning you were given a number. What was it? | 1 | 1310 |  | All other mentions |
| 27 | What exactly did you have to do with this figure? | 2 | Always subtract 13 | Loudly | All other mentions |
| 28 | What did you have to do if you made a mistake? | 1 | Start again from the beginning | 1310 | All other mentions |
| 29 | Did you have to perform another task? If so, which task did you get? | 2 | Yes, sing a song | All my little ducklings | No, all other mentions |
| 30 | Which song did you have to sing? | 1 | All my little ducklings | A children's song | All other mentions |
| 31 | How many verses did the song have? | 1 | 4 |  | All other mentions |
| 32 | Were there animals in the song? If so, which animals appeared? | 5 | Yes: ducks, geese, chicken, pigeons |  | No |
| 33 | Did you have to perform another task? If so, what was your task? | 1 | No |  | Yes, all other mentions |
| *Note.* The 10 questions about the location include 29 answers about the location; the 8 questions about the persons include 9 answers about the persons, and the 15 questions about the situation include 23 answers about the situation. | | | | | |
